# Coupled dynamics modeling and stick-slip suppression of a torque-balanced drilling tool driven by series counter-rotating screw motors

**DOI:** 10.1371/journal.pone.0361858

**Published:** 2026-11-13

**Authors:** Honggang He, Wenjun Chen, Hua Shi, Xin Huang, Li Liu, Yao He, Wei Guo, Jie Deng

**Affiliations:** 1 School of Mechanical and Electrical Engineering, Yibin University, Yibin, Sichuan, China; 2 Sichuan SMT Printing Engineering Technology Research Center, Yibin University, Yibin, Sichuan, China; 3 Yibin Mechanical and Electronic Engineering Technology Research Center, Yibin University, Yibin, Sichuan, China; 4 Research and Development Center, Yibin Sanjiang Machinery Co., Ltd., Yibin, Sichuan, China; King Mongkut’s University of Technology North Bangkok, THAILAND

## Abstract

Excessive drill-string torque and stick-slip vibration severely restrict the safety and efficiency of deep and directional drilling. This paper proposes a torque-balanced drilling tool driven by positive- and reverse-hand screw motors in series, with a buffer airbag innovatively integrated in the middle of the series flow path. A torsional-hydraulic coupled dynamic model containing three rotating subsystems is established by the lumped-parameter method, and a nonlinear rate-weakening bit-rock interaction model is introduced. Modal analysis, frequency-domain analysis and time-domain simulations under step and impulse disturbances are carried out. The results show that the residual drill-string torque is reduced by 66.7% in steady state; the system without airbag has three dominant oscillation modes (1.76, 3.02 and 20.50 Hz); the inner bit is the most disturbance-sensitive component, with a step overshoot of 8.62% and a stick-slip limit cycle under low-speed conditions, while the outer bit remains stable. After introducing the airbag (compliance 5 × 10^−8^ m³/Pa), the impulse pressure-drop spike is attenuated by 51%, the residual speed oscillation by about 82%, and the stick-slip limit-cycle amplitude by more than 96%. The proposed tool provides a new technical approach for downhole torque balancing and vibration suppression in deep wells.

## 1. Introduction

As global oil and gas exploration and development moves toward deep, ultra-deep and unconventional reservoirs, the number of deep wells, ultra-deep wells, extended-reach wells and horizontal wells is increasing [1]. Under these complex well conditions, the drill string works in an extremely harsh environment in the slender borehole. As well depth increases, the drill string can reach several thousand or even ten thousand meters, resulting in a sharp decrease in system stiffness and a significant increase in compliance. Furthermore, reliable downhole flow and injection profiling, as extensively investigated in thermal recovery wellbores [2], provides a critical reference for diagnosing hydraulic conditions and validating the design of downhole flow-distribution systems under extreme environments. Under the combined action of weight on bit (WOB) and torque, the drill string is prone to nonlinear dynamic behaviors such as buckling, lateral vibration and torsional instability [3].

Stick-slip vibration is one of the most destructive phenomena in deep drilling. Its main feature is that the bit experiences periodic “sticking” and “slipping” at the bottom hole: in the sticking phase, the bit speed drops to zero or even reverses, while the drill string is continuously twisted by the surface drive and accumulates elastic potential energy; when the accumulated torque is sufficient to overcome the cutting torque required for rock breaking, the bit suddenly releases the energy, producing an extremely high instantaneous speed, even several times the surface speed [4]. Such severe torsional vibration not only causes bit tooth breakage and premature bearing failure, but also leads to fatigue damage of the drill string, seriously reduces the rate of penetration (ROP), and may even cause catastrophic accidents such as stuck pipe and tool failure [5].

In recent years, the modeling of drill-string vibration has developed from lumped-parameter models to distributed-parameter models. Ghasemloonia et al. [6] systematically reviewed the modeling and suppression methods of drill-string vibration, pointing out that the coupling effect of axial, torsional and lateral vibrations is the fundamental reason for the intensification of vibration in deep drilling. Castello and Ritto [7] performed model selection and parameter estimation of bit-rock interaction models based on a Bayesian approach, and found that the uncertainty of the rate-weakening exponent has a significant impact on stick-slip prediction. Tengesdal et al. [8] proposed a component-based nonlinear dynamics modeling method for drill strings, which effectively improves the prediction accuracy of vibration in complex well conditions. Goicoechea et al. [9] established a Cosserat rod drill-string model considering the arbitrariness of borehole geometry, which can simultaneously capture the bending, torsional and axial deformation of the drill string. However, the above studies mostly focus on traditional single-bit drive systems. For multi-power-source coupled systems containing downhole power drilling tools (such as positive displacement motors, PDM), dynamics modeling still faces challenges. Jiao et al. [10] pointed out that PDM has become the standard configuration for deep and ultra-deep well drilling, but existing drill-string dynamics models often ignore the influence of PDM on axial-torsional coupled vibration, resulting in deviations in field stick-slip prediction. In addition, thermal‑mechanical coupling effects in downhole tubular systems have been shown to significantly influence structural integrity under extreme conditions [11], highlighting the need for multi‑physics considerations in tool design.

At present, the main approaches to mitigate stick-slip vibration include active control (such as adjusting the top-drive speed) and passive suppression (such as using shock absorbers). However, in long well sections, the signal transmission of surface control has a serious hysteresis effect, making it difficult to respond to the instantaneous conditions at the bottom hole in real time. Therefore, the development of torque-balancing and vibration suppression technologies based on downhole tools has important engineering application value.

Drill-string dynamics has developed from early single-mode vibration to multi-degree-of-freedom coupled vibration. Castello and Ritto [7] systematically studied bit-rock interaction models and their stability, revealing the mechanism by which rate-weakening friction induces stick-slip self-excited oscillation. Tian et al. [12,13] systematically revealed the generation mechanism and suppression methods of drill-string stick-slip vibration through motion simulation and experimental research. Faghihi et al. [14] established an axial-torsional coupled dynamics control model for distributed drilling systems, which effectively suppressed stick-slip vibration through boundary control. Auriol et al. [5] compared the effects of various advanced control strategies (including delayed feedback control, velocity feedback control and torque feedback control) in eliminating stick-slip oscillation of drill strings, and pointed out that control methods based on the propagation characteristics of torsional waves in the drill string have better robustness. The above studies provide a theoretical basis for active control of drill-string vibration, but in long well sections, the transmission delay of control signals and the reliability of downhole actuators are still the bottlenecks of engineering applications. In other engineering domains where strong, fast-varying disturbances must likewise be rejected in real time, adaptive disturbance-compensation control has recently achieved notable progress, e.g., modified super-twisting sliding mode control with adaptive gain scheduling [15] and adaptive anti-skid braking control with runway disturbance identification [16]; these advances further motivate the disturbance-rejection-oriented downhole tool design pursued in this work.

In order to fundamentally reduce drill-string torque, scholars at home and abroad have proposed a variety of torque-balanced drilling tool schemes. Li et al. [17] studied an electrically driven torque-balancing system, and Guo et al. [18] designed and optimized a linear rotary drilling motor, but such schemes rely on complex downhole power supply and control systems, and their reliability faces challenges in the high-temperature and high-pressure environment of deep wells. Recently, He et al. [19] proposed a novel dual-output torque-balanced drilling tool based on planetary motion and investigated its coupled dynamics and stick-slip suppression. The mechanically driven dual-bit system uses gearboxes for direction reversal, and the gears are prone to wear under high downhole loads, resulting in low efficiency [20]. The series screw-motor drive system uses positive displacement motors (PDM) as the power source, is fully hydraulically driven, and has good high-temperature and high-pressure resistance, but existing studies mostly focus on the dynamic characteristics of single-stage screw motors [21], and the fluid-solid coupling mechanism under the series architecture of positive- and reverse-hand screw motors has not been deeply revealed. Gao et al. [22] proposed a downhole dual-bit torque self-balancing drilling system, which drives the inner and outer bits to rotate synchronously in opposite directions through a downhole power assembly, and adjusts the WOB of the inner and outer bits through a pressure regulation system to achieve mutual torque balancing downhole, thereby offsetting the reaction torque generated by drill-string rotation in conventional drilling. Laboratory tests show that the system has good adaptability to different lithologies and little disturbance to the borehole wall. However, the system relies on an independent downhole motor drive, and its long-term reliability in high-temperature and high-pressure environments still needs to be verified. Tikhonov and Bukashkina [23] established a complete three-dimensional drill-string dynamics model including PDM, bit and rock, and found that PDM excitation is one of the important factors inducing high-frequency torsional oscillation (HFTO), but the model does not consider the fluid-solid coupling effect when multiple screw motors are driven in series.

In terms of hydraulic pulsation suppression, accumulators (airbags) are often used to absorb pressure pulsations. Kondrat’eva and Popov [24] experimentally studied the attenuation of pressure pulsations by hydraulic accumulators, and found that the volume and pre-charge pressure of the accumulator are the key parameters affecting the suppression effect. In oil and gas drilling, a pulsation damper is often installed at the outlet of the mud pump to stabilize the flow and pressure of the drilling fluid. Its working principle is similar to that of an airbag accumulator, absorbing the periodic fluctuation of the pump displacement through the elastic deformation of a compressible gas (usually nitrogen) [24]. However, existing studies mostly apply accumulators to surface mud pump systems or simple hydraulic circuits, and research on integrating them into the middle flow path of downhole series screw drilling tools to decouple the hydraulic coupling between the upper and lower screw motors is still blank. Recent studies on fluid-structure interaction in confined channels have demonstrated that passive compliance elements can effectively attenuate high-frequency pressure fluctuations [25], providing a theoretical basis for the airbag configuration proposed in this work.

To address these issues, this paper proposes a novel torque-balanced drilling tool driven by series positive- and reverse-hand screw motors, featuring an integrated buffer airbag in the flow path. Based on fluid mechanics and rotor dynamics, a fluid-solid coupling dynamic model is established, incorporating nonlinear rock interactions and high-order fluid network equations. Using realistic geometric parameters from a deep-well screw drilling tool, numerical simulations are conducted to comparatively analyze the system’s dynamic characteristics with and without the airbag. Finally, a collaborative optimization of mechanical-hydraulic parameters is performed based on sensitivity analysis.

## 2. Materials and methods

### 2.1. System structure and torque balancing principle

The drilling tool system is mainly composed of an upper screw assembly, an intermediate adapter assembly (integrated with a buffer airbag), a lower screw assembly and a dual-bit assembly (as shown in Fig 1 and Fig 2). The upper screw is a positive-hand screw motor, whose rotor outputs power through a transmission shaft; the rotor of the upper screw drilling tool is connected through this transmission shaft and drives the housing of the lower screw motor to rotate. The lower screw is a reverse-hand screw motor, whose housing is connected to the outer bit and whose rotor is connected to the inner bit. An annular buffer airbag is arranged at the middle connecting flow path between the upper and lower screw motors; the flow path remains unobstructed, and the airbag is filled with a compressible gas (such as nitrogen) to absorb the pressure pulsation of the power fluid through volume change.

**Fig 1:**
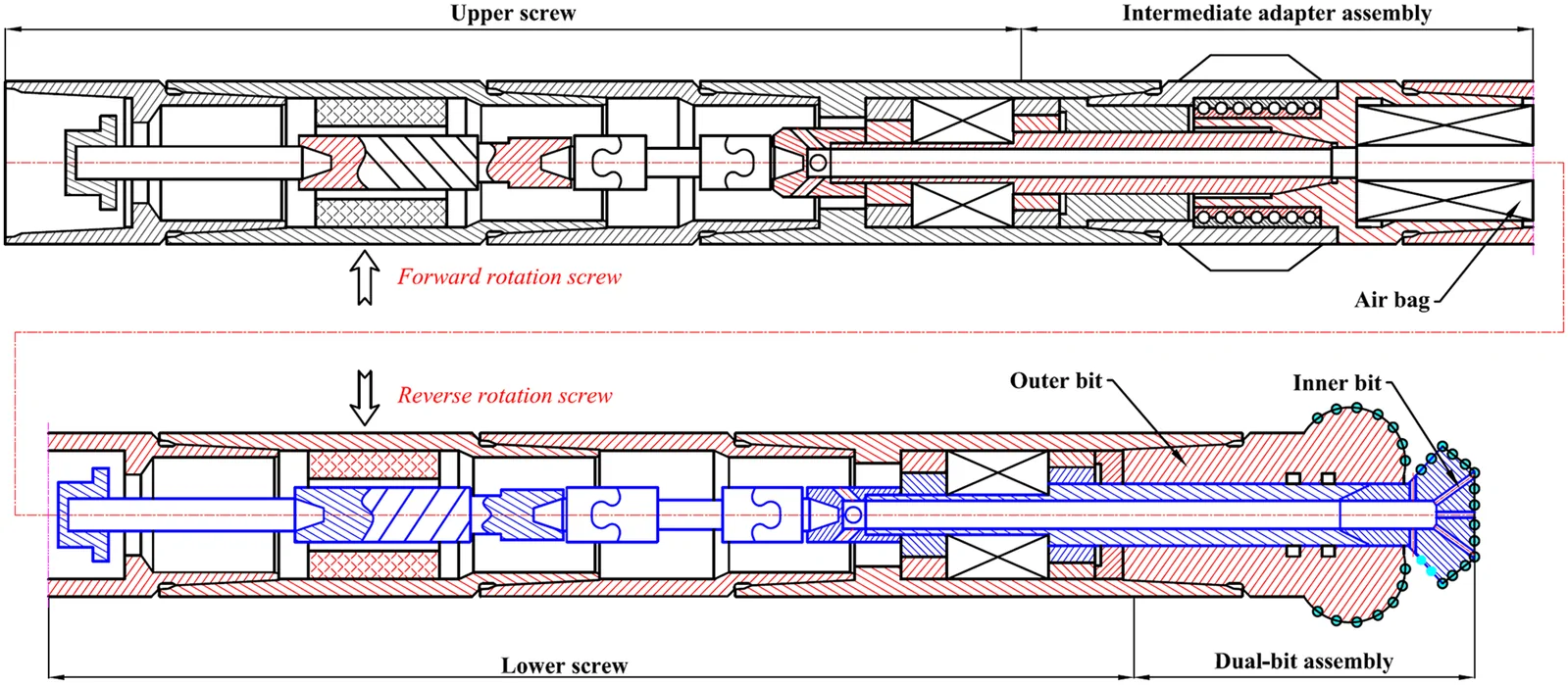
Structural schematic of the torque-balanced drilling tool.

**Fig 2:**
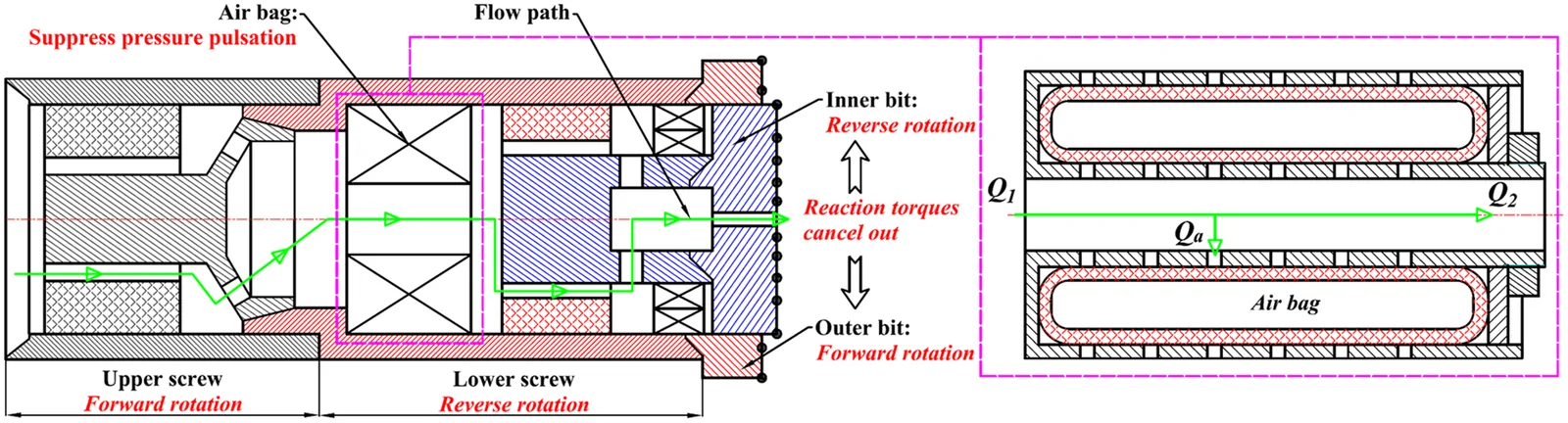
Working principle of the torque-balanced drilling tool.

The upper and lower screw motors are supplied with drilling fluid in series and share the same flow rate *Q*. For a positive displacement screw motor, the speed of the rotor relative to the stator is proportional to the flow rate [21]:


$$\begin{array}{c} Q=V_{i}\omega_{i,rel} \end{array}$$ (1)

where *V*_i_ is the displacement per radian and *ω*_i,rel_ is the speed of the rotor relative to the stator. Let the displacement of the upper screw be V_1_ and that of the lower screw be V_2_, with V_1_/V_2_ = η = 2. Based on the topology of fluid transmission and mechanical connection, the kinematic derivation is as follows.

Speed of the upper screw rotor:


$$\begin{array}{c} \omega_{\text{1}}=\frac{Q}{V_{\text{1}}} \end{array}$$ (2)

Speed of the lower screw housing (i.e., the outer bit speed): since the upper screw rotor mechanically drives the lower screw housing through the transmission shaft, the two speeds are equal (neglecting the elastic deformation of the transmission shaft):


$$\begin{array}{c} \omega_{outer}=\omega_{\text{2}}=\omega_{\text{1}} \end{array}$$ (3)

Relative speed of the lower screw rotor: after the fluid enters the lower screw, it drives the lower screw rotor to rotate in the opposite direction relative to the housing:


$$\begin{array}{c} \omega_{rel}=\frac{Q}{V_{\text{2}}}=\eta \omega_{\text{1}} \end{array}$$ (4)

Absolute speed of the inner bit: the absolute speed of the lower screw rotor (inner bit) is the superposition of the housing speed and the relative reverse speed:


$$\begin{array}{c} \omega_{inner}=\omega_{outer}-\omega_{rel}=(\text{1}-\eta )\omega_{\text{1}} \end{array}$$ (5)

When η = 2, *ω*_inner_ = −ω_1_. Thus, the outer bit and the inner bit rotate at equal speeds in opposite directions. If the rock-breaking torques are designed to satisfy *T*_outer_ + *T*_inner_ ≈ 0, the residual torque transmitted to the drill string approaches zero.

It should be emphasized that Equation (3) is a steady-state kinematic relation used only to derive the speed-matching condition, not a rigid constraint imposed on the dynamic model. In the coupled model of Section 2.2, the universal transmission shaft is explicitly represented as an elastic element with torsional stiffness *k*_**1**_ and damping *c*_**d1**_ (Equation (10)); the twist *θ*_**1**_ − *θ*_**2**_ is itself a state variable (Equations (22) and (23)), so the torsional flexibility between the upper rotor and the lower housing is fully retained in all subsequent dynamic analyses. Backlash of the universal joint is neglected because the tool operates under a unidirectional torque preload by design: the residual torque *τ*_**1**_ = 300 N·m is intentionally kept positive (Section 3.1), so that the joint flanks remain permanently engaged on the same contact side and the transmission never traverses the backlash dead zone, which would only become relevant under torque reversal.

### 2.2. Coupled dynamics modeling

To simplify the model for analytical and numerical solution, the following assumptions are made:

(1) Each rotating component is simplified as a rigid body with lumped moment of inertia;
(2) The mass of the transmission shaft is neglected, and it is simplified as an elastic element with torsional stiffness and damping;
(3) The drilling fluid is regarded as a compressible Newtonian fluid, considering its fluid inertia, flow resistance and compressibility;
(4) The output torque of the screw motor is linearly related to the inlet-outlet pressure difference;
(5) Lateral and axial vibrations are not considered for the time being, and only the coupling of the torsional degree of freedom and fluid pressure is studied. The weight on bit is likewise treated as quasi-static over the fast torsional-hydraulic transients studied here; the implications of dynamic WOB fluctuations and axial-torsional coupling are addressed in Section 4.4.

To describe the torsional vibration characteristics of the system, based on the lumped-parameter method, the upper screw rotor, the lower screw housing (including the outer bit) and the lower screw rotor (including the inner bit) are simplified as three rotating rigid bodies with lumped moments of inertia. The generalized coordinates of the system are defined as:

θ_1_: angular displacement of the upper screw rotor;
θ_2_: angular displacement of the lower screw housing (i.e., the outer bit);
θ_3_: angular displacement of the lower screw rotor (i.e., the inner bit).

Based on Newton’s second law, the forces on the above three rotating bodies are analyzed respectively, and their torsional dynamic differential equations are established [8]:

(1) Upper screw rotor (J_1_):

$$\begin{array}{c} J_{\text{1}}{\ddot{\theta }}_{\text{1}}=T_{\text{1}}-c_{\text{1}}{\dot{\theta }}_{\text{1}}-\tau_{\text{1}} \end{array}$$ (6)

(2) Lower screw housing and outer bit (J_2_):

$$\begin{array}{c} J_{\text{2}}{\ddot{\theta }}_{\text{2}}=\tau_{\text{1}}+T_{\text{2}}-c_{\text{2}}{\dot{\theta }}_{\text{2}}-T_{outer} \end{array}$$ (7)

(3) Lower screw rotor and inner bit (J_3_):

$$\begin{array}{c} J_{\text{3}}{\ddot{\theta }}_{\text{3}}=-T_{\text{2}}-c_{\text{3}}\left({\dot{\theta }}_{\text{3}}-{\dot{\theta }}_{\text{2}}\right)-T_{inner} \end{array}$$ (8)

In the above equations, the physical meanings and expressions of the core parameters are as follows [25]:


$$\begin{array}{c} T_{\text{1}}=\frac{V_{\text{1}}\Delta p_{\text{1}}}{\text{2}\pi },\;T_{\text{2}}=\frac{V_{\text{2}}\Delta p_{\text{2}}}{\text{2}\pi } \end{array}$$ (9)

where τ_1_ is the torque transmitted by the transmission shaft connecting the upper screw rotor and the lower screw housing. Simplifying it as an elastic element with torsional stiffness and damping, its constitutive relation is [21]:


$$\begin{array}{c} \tau_{\text{1}}=k_{\text{1}}\left(\theta_{\text{1}}-\theta_{\text{2}}\right)+c_{d\text{1}}\left({\dot{\theta }}_{\text{1}}-{\dot{\theta }}_{\text{2}}\right) \end{array}$$ (10)

Equation (10) is a linearization of the universal-shaft torque about the rated operating point. A real downhole universal joint exhibits nonlinear geometric limits: as the bending angle *β* increases, the transmitted torque becomes angle-dependent, a second-order speed fluctuation of relative magnitude 1 − cos *β* appears between the input and output shafts, and contact constraints bound the maximum allowable deflection. These effects are neglected here because the centralizer-guided configuration of Fig 1 keeps the operating bending angle below approximately 3°, at which the kinematic speed error of the joint is below 0.2% and its torque-angle characteristic is practically linear. Incorporating the full nonlinear joint kinematics and its geometric limits is identified as future work in Section 4.4.

The drilling fluid flows through the pump-pipeline-upper screw-intermediate flow path-lower screw-bit, forming a series hydraulic network.

#### 2.2.1. Condition without Airbag (Base Model).

Without considering the airbag, the system flows through a single pipeline and the flow rate is equal everywhere. According to the fluid continuity equation and momentum equation [24]:


$$\begin{array}{c} \frac{d\left(\Delta p_{\text{1}}\right)}{dt}=\frac{Q-V_{\text{1}}{\dot{\theta }}_{\text{1}}}{C_{f}} \end{array}$$ (11)

$$\begin{array}{c} \frac{d\left(\Delta p_{\text{2}}\right)}{dt}=\frac{Q-V_{\text{2}}\left({\dot{\theta }}_{\text{2}}-{\dot{\theta }}_{\text{3}}\right)}{C_{f}} \end{array}$$ (12)

$$\begin{array}{c} \frac{dQ}{dt}=\frac{P_{pump}-\Delta p_{\text{1}}-\Delta p_{\text{2}}-R_{f}Q}{L_{f}} \end{array}$$ (13)

#### 2.2.2. Condition with Airbag (Improved Model).

A buffer airbag is added to the connecting flow path between the upper and lower screw motors. The airbag divides the flow path into an upstream section (flow rate Q_1_) and a downstream section (flow rate Q_2_). According to the flow conservation at the node [24]:


$$\begin{array}{c} Q_{\text{1}}=Q_{\text{2}}+Q_{a} \end{array}$$ (14)

The relationship between the fluid volume change in the airbag and the airbag pressure is equivalent to a fluid capacitance [24]:


$$\begin{array}{c} Q_{a}=C_{a}\frac{dp_{a}}{dt} \end{array}$$ (15)

The fluid momentum equations and pressure-drop dynamic equations of the two pipeline sections are modified as:


$$\begin{array}{c} \frac{dQ_{\text{1}}}{dt}=\frac{P_{pump}-\Delta p_{\text{1}}-p_{a}-R_{f\text{1}}Q_{\text{1}}}{L_{f\text{1}}} \end{array}$$ (16)

$$\begin{array}{c} \frac{dQ_{\text{2}}}{dt}=\frac{p_{a}-\Delta p_{\text{2}}-R_{f\text{2}}Q_{\text{2}}}{L_{f\text{2}}} \end{array}$$ (17)

$$\begin{array}{c} \frac{d\left(\Delta p_{\text{1}}\right)}{dt}=\frac{Q_{\text{1}}-V_{\text{1}}{\dot{\theta }}_{\text{1}}}{C_{f\text{1}}} \end{array}$$ (18)

$$\begin{array}{c} \frac{d\left(\Delta p_{\text{2}}\right)}{dt}=\frac{Q_{\text{2}}-V_{\text{2}}\left({\dot{\theta }}_{\text{2}}-{\dot{\theta }}_{\text{3}}\right)}{C_{f\text{2}}} \end{array}$$ (19)

Based on experimental and modeling studies of bit-rock interaction [7], the resisting torques of the inner and outer bits can be uniformly simplified as an exponential rate-weakening model:


$$\begin{array}{c} T_{bit}(\omega )=T_{ref}{\left(\frac{\left|\omega \right|}{\omega_{ref}}\right)}^{-\gamma } \end{array}$$ (20)

To reveal the negative damping mechanism of stick-slip vibration, Equation (20) is expanded in a Taylor series near the steady speed, and the first-order linear term is taken [5]. The equivalent damping is:


$$\begin{array}{c} c_{eq}=-\gamma \frac{T_{ref}}{\omega_{ref}}{\left(\frac{\omega_{\text{0}}}{\omega_{ref}}\right)}^{-\gamma -\text{1}} \end{array}$$ (21)

Since *T*_ref_ > 0 and γ > 0, *c*_eq_ < 0 [7], that is, the system introduces “negative damping,” which easily evolves into sustained periodic oscillation.

For the system without airbag, a 7-dimensional state vector is defined:


$$\begin{array}{c} {𝐱}_{no}={\left[\theta_{\text{1}}-\theta_{\text{2}},{\dot{\theta }}_{\text{1}},{\dot{\theta }}_{\text{2}},{\dot{\theta }}_{\text{3}},\Delta p_{\text{1}},\Delta p_{\text{2}},Q\right]}^{T} \end{array}$$ (22)

The lumped‑parameter modeling approach adopted here is complemented by recent advances in poromechanical coupling [26], which inform the treatment of fluid‑solid interactions in the series‑connected dual‑motor topology.

For the system with airbag, a 9-dimensional state vector is defined:


$$\begin{array}{c} {𝐱}_{ab}={\left[\theta_{\text{1}}-\theta_{\text{2}},\;{\dot{\theta }}_{\text{1}},\;{\dot{\theta }}_{\text{2}},\;{\dot{\theta }}_{\text{3}},\Delta p_{\text{1}},\Delta p_{\text{2}},p_{a},Q_{\text{1}},Q_{\text{2}}\right]}^{T} \end{array}$$ (23)

The input vector is u = [*T*_inner_]^T^, and the system can be expressed as ẋ = Ax + Bu, which lays the foundation for subsequent stability analysis.

### 2.3. Determination of system parameters

As shown in Table 1, the parameter values are mainly based on reference [25] and the drill-string dynamics standard handbook [28], determined on the basis of a commonly used 5LZ172×7.0 screw drilling tool for deep wells. The selection of bit-rock interaction parameters also references recent experimental calibrations of impregnated diamond bits under varying wear states [29], which provide a basis for the rate-weakening exponent used in this study.

**Table 1:** System dynamics parameters.

Parameter category	Parameter name	Symbol	Value	Unit	Source and description
Inertia	Upper screw rotor inertia	J_1_	5.0	kg·m²	Estimated from rotor geometry [25]
Inertia	Lower screw housing + outer bit inertia	J_2_	8.0	kg·m²	Estimated from motor housing and bit dimensions [25]
Inertia	Lower screw rotor + inner bit inertia	J_3_	3.0	kg·m²	Estimated from rotor and small inner bit
Stiffness & damping	Transmission shaft torsional stiffness	k_1_	5.0 × 10^4^	N·m/rad	Comprehensive stiffness of universal shaft and transmission shaft [27]
Stiffness & damping	Transmission shaft torsional damping	*c*_d1_	200	N·m·s/rad	Empirical value of material internal damping and structural damping
Stiffness & damping	Bearing viscous damping	c_1_/c_2_/c_3_	50/80/40	N·m·s/rad	Characteristics of screw motor rubber bearings [25]
Hydraulic	Upper screw displacement	V_1_	0.010	m³/r	Corresponding to a 5-stage motor, 1.0 L per revolution [25]
Hydraulic	Lower screw displacement	V_2_	0.005	m³/r	Set displacement ratio η = 2
Hydraulic	Pipeline flow resistance	*R*_f_	1.0 × 10^8^	Pa·s/m³	Calculated from friction losses
Hydraulic	Pipeline fluid inertance	*L*_f_	5.0 × 10^7^	Pa·s²/m³	Estimated from the fluid column inertia of the pipeline
Hydraulic	Fluid compressibility compliance	*C*_f_	1.0 × 10^−9^	m³/Pa	Considering fluid bulk modulus and pipeline elasticity [27]
Hydraulic	Airbag compliance	*C*_a_	5.0 × 10^−8^	m³/Pa	Improved design parameter, about 50 times *C*_f_

Note: The displacements V_1_ and V_2_ in the table are in m³/r (displacement per revolution); in the theoretical derivation the displacement is in m³/rad (displacement per radian), and the unit conversion V/(2π) is applied in numerical calculation.

### 2.4. Numerical calculation procedure

Since the model contains hydraulic capacitive elements with small compliance and mechanical rotating bodies with large inertia, the system presents obvious stiffness characteristics. If a conventional explicit Runge-Kutta solver is used, it is easy to diverge or take too long. Therefore, a variable-step stiff solver based on backward differentiation formulas (BDF) is selected for time-domain integration, with the relative error tolerance set to 10^−4^ and the maximum step size limited to 0.01 s, so as to ensure the synchronous convergence of high-frequency hydraulic pulsation and low-frequency mechanical torsion. All author-generated Python code used to produce the results of this study is provided as Supporting Information (S1 File) and will be made publicly available without restrictions upon publication.

The whole simulation is divided into three stages: steady-state initialization, modal frequency-domain analysis and time-domain transient analysis. The specific procedure is as follows:

(1) Steady-state operating point solution: set all time-derivative terms in Equations (6)-(19) to zero, input a constant pump pressure *P*_pump_ and the reference rock-breaking torque, and solve the nonlinear algebraic equations by the Newton iteration method to obtain the initial state vector x₀.
(2) Jacobian matrix and modal calculation: linearize the nonlinear equations at x₀ and extract the state matrix A. The eigenvalues and eigenvectors of A are then computed, and the natural frequencies and damping ratios of the system without airbag (7th order) and with airbag (9th order) are analyzed
(3) Frequency-domain response calculation: construct the transfer function based on the state-space model, and the frequency-domain characteristics are obtained by evaluating the resolvent (jωI − A)⁻¹B over a logarithmic frequency grid from the inner-bit load disturbance to each state output of the system.
(4) Time-domain transient integration: substitute the nonlinear rock interaction model (Equation (20)) into the dynamic equations to construct the ODE function with nonlinear disturbance. Apply step load (simulating a sudden change in formation hardness) and impulse load (simulating bit bounce), and the transient responses are integrated with the stiff solver.
(5) Post-processing and evaluation: extract the time-domain response data under each condition, and calculate the speed overshoot σ = (*ω*_max_ − ω_∞)/|ω_ ∞ | × 100%, the peak pressure spike and the settling time.

## 3. Results

### 3.1. Steady-state characteristics and modal analysis

This section reveals the inherent characteristics of the base system without a buffer airbag and its response laws under disturbances through modal analysis and time-domain simulation.

Solving the steady-state operating point: set the pump pressure *P*_pump_ = 10 MPa, and obtain the upper screw speed ω_1_ = ω_2_ = 31.1146 rad/s and the inner bit speed ω_3_ = −31.1146 rad/s. Set the outer-bit rock-breaking torque *T*_outer_ = 900 N·m and the inner-bit reverse torque *T*_inner_ = −600 N·m, then the residual drill-string torque τ_1_ = 300.0 N·m. Compared with the traditional single bit (residual torque up to 900 N·m), the torque is reduced by 66.7%.

Notably, the forward torque of the outer bit (900 N·m) is intentionally designed to exceed the reverse torque of the inner bit (600 N·m). This ensures a net residual torque in the make-up direction, maintaining the drill string in a continuously preloaded state. Such a design effectively prevents back-off failures of the bottomhole assembly (BHA) under dynamic vibrations, thereby enhancing operational safety and reliability.

The state matrix is decomposed by eigenvalues, and three dominant oscillation modes are identified:

Mode 1 (1.76 Hz, damping ratio 0.392): inner-bit-fluid coupled vibration. Since the inner bit has a small inertia and is affected by kinematic amplification, it is easily excited by low-frequency disturbances.

Mode 2 (3.02 Hz, damping ratio 0.350): lower-screw fluid-mechanical coupled vibration, manifested as the mutual feedback between the pressure drop Δp_2_ and the inner-bit speed.

Mode 3 (20.50 Hz, damping ratio 0.291): transmission shaft torsional vibration; high-frequency resonance will aggravate the fatigue of the transmission shaft.

The frequency response characteristics of the system without airbag are shown in Fig 3. Three obvious resonance peaks can be observed from the Bode diagram, corresponding to the above three dominant oscillation modes.

**Fig 3:**
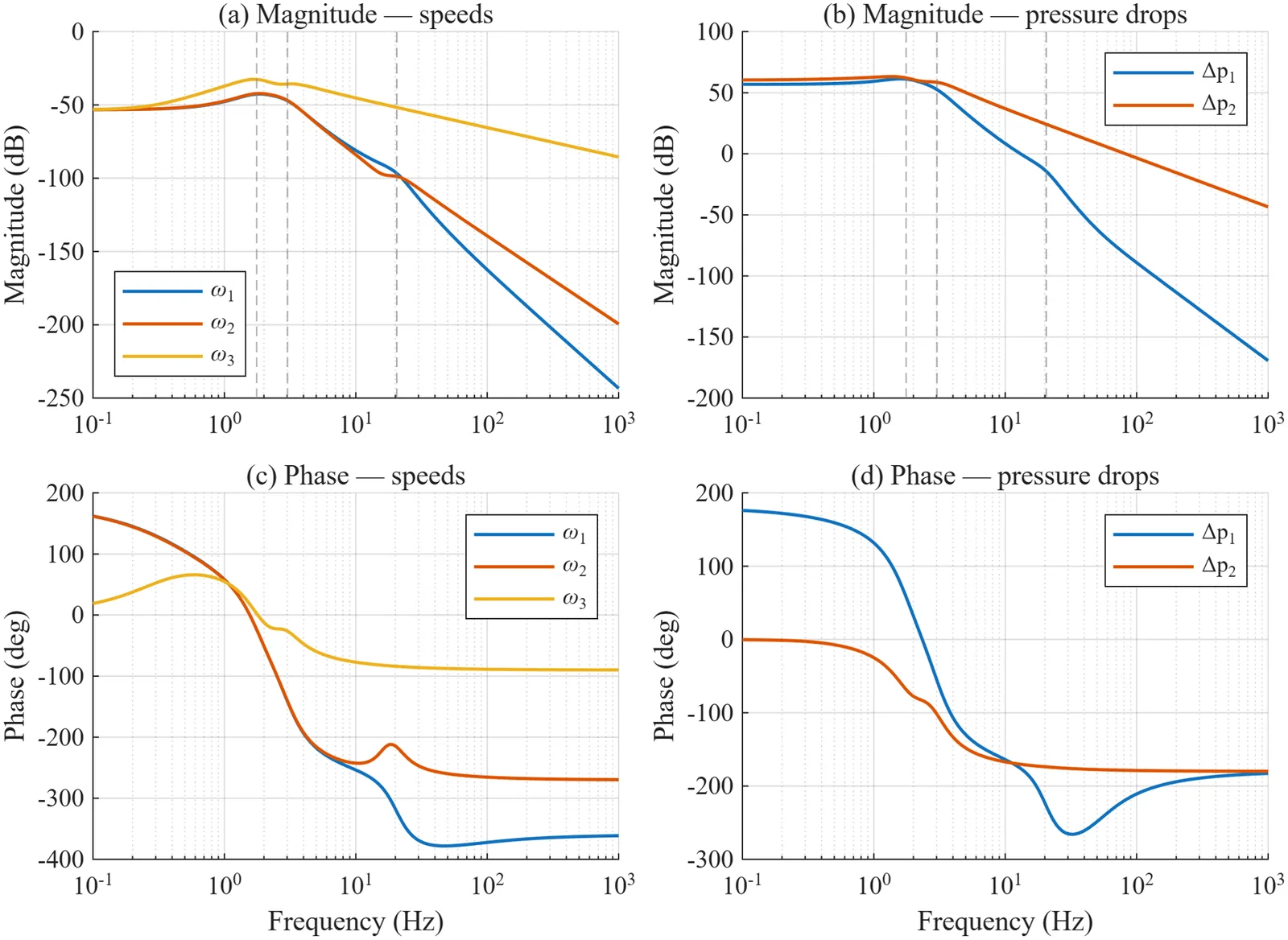
Frequency response of the system without airbag.

To complement the modal analysis, a formal local controllability and observability analysis was performed on the linearized state-space models at the steady-state equilibrium *x*_**0**_ (10 MPa rated condition). Taking the inner-bit load disturbance as the input *u* and the three rotational speeds (*ω*_**1**_, *ω*_**2**_, *ω*_**3**_) as the measured outputs, the Kalman controllability matrix [*B AB* … *A*^n-1^*B*] and the observability matrix [*C*^T^ (*CA*)^T^ … (*CA*^n-1^)^T^]^T^ were evaluated for the 7-state model without airbag (Equation (22)) and the 9-state model with airbag (Equation (23)). Both matrices are full rank for the two systems (rank 7 of 7 and rank 9 of 9, respectively; the rank tests were carried out with state scaling because the stiff, multi-timescale dynamics make the unscaled matrices ill-conditioned). The two systems are therefore locally controllable and locally observable around the operating point. Notably, full observability is retained even when only the inner-bit speed *ω*_**3**_ is measured, indicating that the onset of the inner-bit stick-slip instability identified above can in principle be detected and reconstructed from a single downhole speed measurement.

### 3.2. Linear time-domain dynamic responses

(1) Step load response

At t = 0.5 s, the inner-bit rock-breaking torque is stepped up by 200 N·m (simulating a hard interlayer; the amplitude is selected according to the anti-back-off constraint to ensure that the total torque of the inner bit does not exceed the outer-bit torque of 900 N·m). The simulation shows that the maximum deviation of the inner-bit speed ω_3_ is 2.682 rad/s, an overshoot of about 8.62% relative to the steady-state value, which is the highest dynamic sensitivity among all components. The lower-screw pressure drop Δp_2_ rises by about 0.21 MPa in a step to provide additional driving torque; the residual torque decreases from 300 N·m to 100 N·m (transient minimum of about 29 N·m), and the system always remains tightened without back-off.

(2) Impulse load response

An impulse load with an amplitude of 200 N·m and a duration of 0.15 s is applied. The peak speed deviation of the inner bit reaches 2.68 rad/s; the lower-screw pressure drop Δp_2_ spikes to 0.228 MPa (about 2.3% of the pump pressure), and all states quickly return to steady state after the impact.

The time-domain dynamic response curves of the system without airbag under free vibration, step load and impulse load are shown in Fig 4.

**Fig 4:**
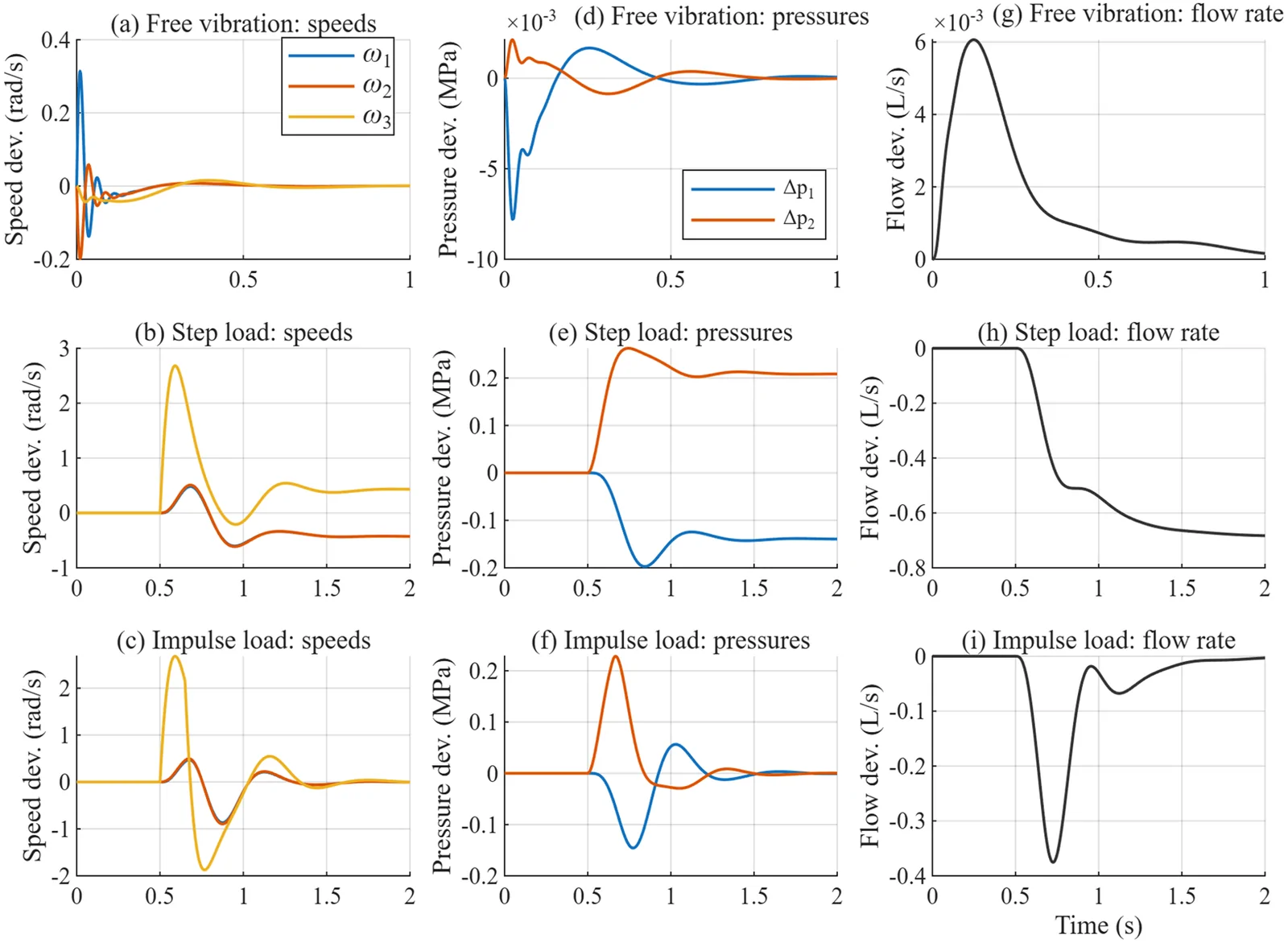
Dynamic response of the system without airbag.

It should be noted that T_1_ and T_2_ are the fluid driving torques of the screw motors, most of which are used to overcome the viscous friction of the screw motor rubber bearings at the rated speed (c_1_ω_1_ ≈ 1556 N·m, c_2_ω_2_ ≈ 2489 N·m); after deducting the friction loss, the effective torque transmitted by the upper screw through the transmission shaft is τ_1_ = 300 N·m, and the effective net torque of the lower screw driving the dual bits to break rock is 600 N·m. The torque distribution and balance characteristics under various loading conditions are shown in Fig 5, in which T_1_ and T_2_ are decomposed into effective torque and viscous friction torque in Fig 5(a).

**Fig 5:**
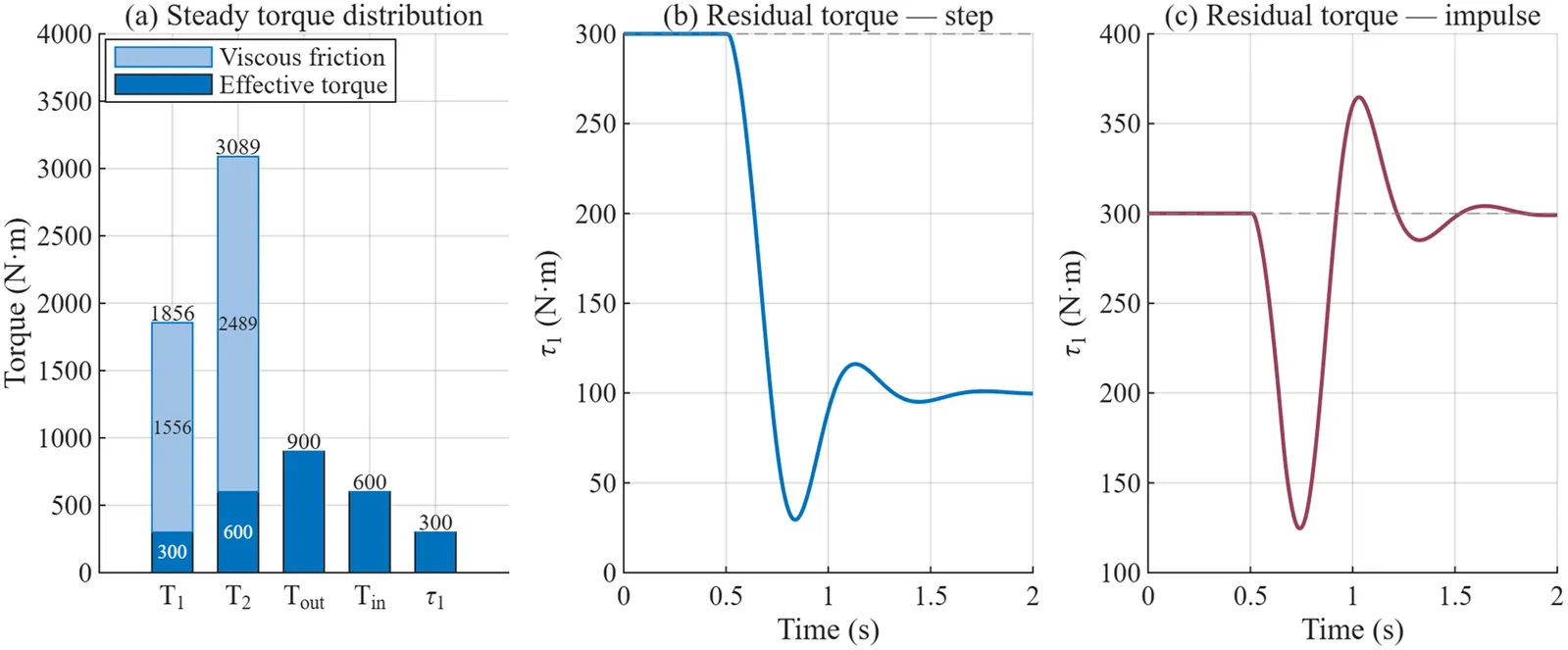
Torque distribution and balance characteristics under various loading conditions.

(3) Outer-bit step load response

At t = 0.5 s, the outer-bit rock-breaking torque is stepped up by 200 N·m (equal amplitude to the inner-bit condition). The simulation shows that the maximum deviation of the outer-bit speed ω_2_ is only 0.680 rad/s (relative deviation of about 2.19%), and the relative deviations of the upper screw and inner-bit speeds are about 2.15% and 2.35% respectively; the transient spike of the upper-screw pressure drop Δp_1_ is about 0.14 MPa; the residual torque τ_1_ increases from 300 N·m to 500 N·m, and the tightening state of the system is further enhanced. The outer-bit disturbance is directly transmitted to the pump source through the upper screw flow path, and the response decays rapidly; its speed deviation is less than one third of that in the inner-bit condition (2.68 rad/s).

(4) Outer-bit impulse load response

An impulse load with an amplitude of 200 N·m and a duration of 0.15 s is applied to the outer bit. The peak speed deviation of the outer bit is about 0.680 rad/s, and the speed deviation of the inner bit is about 1.04 rad/s; the upper-screw pressure drop Δp_1_ spikes to about 0.12 MPa (about 1.2% of the pump pressure), and all states quickly return to steady state after the impact without residual oscillation. Comparing (1), (2) with (3), (4), it can be seen that under equal-amplitude disturbances, the peak speed deviation of the inner bit (2.68 rad/s) is about four times that of the outer bit (0.680 rad/s), and the dynamic sensitivity of the inner bit to external load disturbances is much higher than that of the outer bit.

The deviation responses of the system states relative to steady-state values under outer-bit load disturbances are shown in Fig 6.

**Fig 6:**
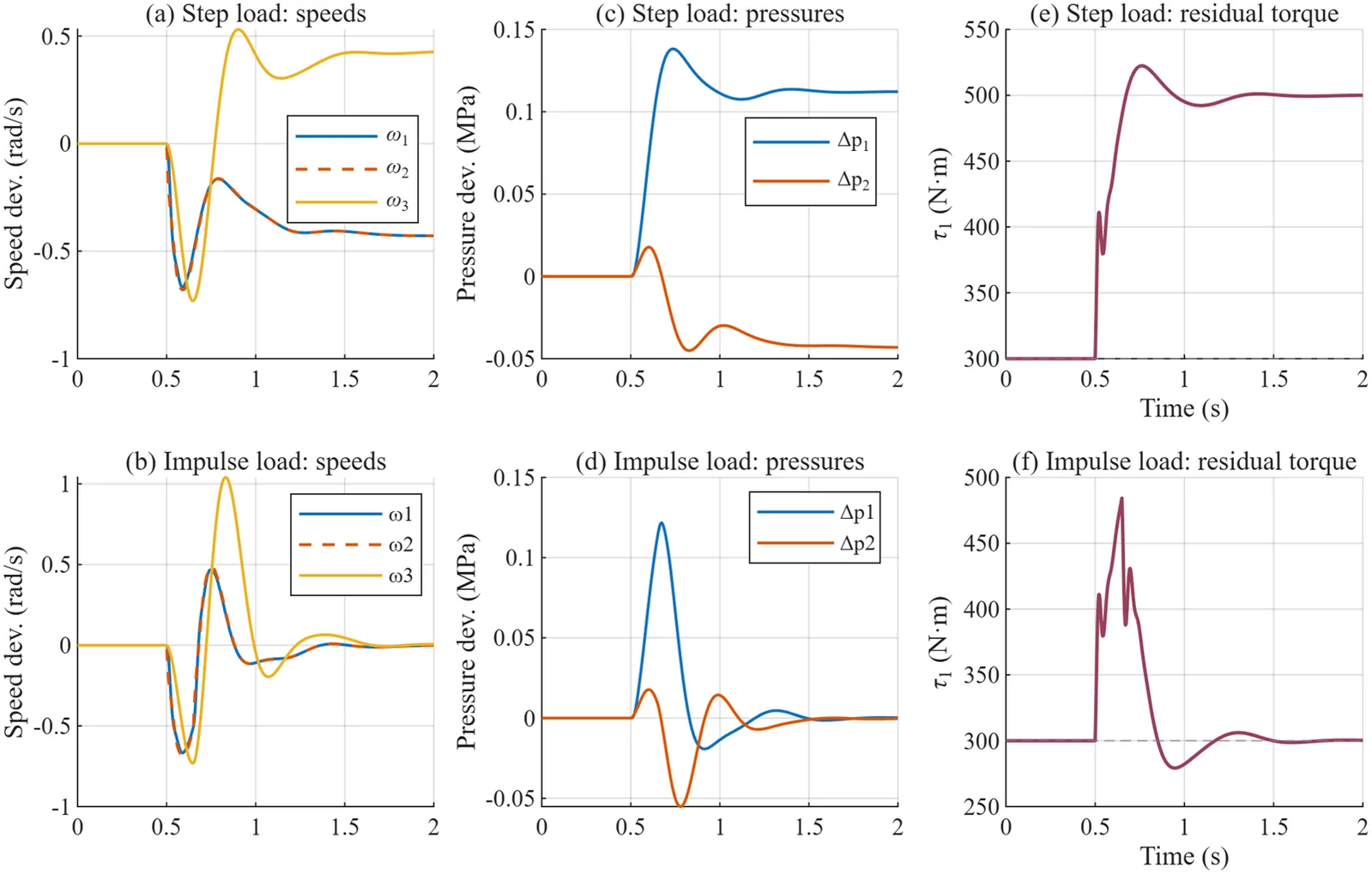
Deviation responses of system states under outer-bit load disturbances.

### 3.3. Nonlinear stick-slip vibration responses

The nonlinear model (Equation (20), γ = 0.3) is substituted, and an initial speed disturbance of −1 rad/s is applied to the inner bit under a low-speed condition (pump pressure 2 MPa, steady-state speed about 3.6328 rad/s). Due to the negative damping effect (*c*_eq_ < 0), the inner bit fails to converge to the steady state, but shows obvious speed fluctuation: the speed periodically oscillates between near zero (sticking) and high speed (slipping), forming a limit cycle with a peak-to-peak value of about 14.5 rad/s and a frequency of about 1.5 Hz, and the bit-rock torque fluctuates violently in the range of 807–1474 N·m. The full-state Jacobian linearization at the equilibrium point of this condition shows two eigenvalues with positive real parts (2.25 1/s and 53.18 1/s), indicating that the system is locally unstable. This shows that in the face of strong nonlinear rock loads, the inner bit is still very prone to stick-slip oscillation.

The comparison of stick-slip vibration under linear and nonlinear models is shown in Fig 7.

**Fig 7:**
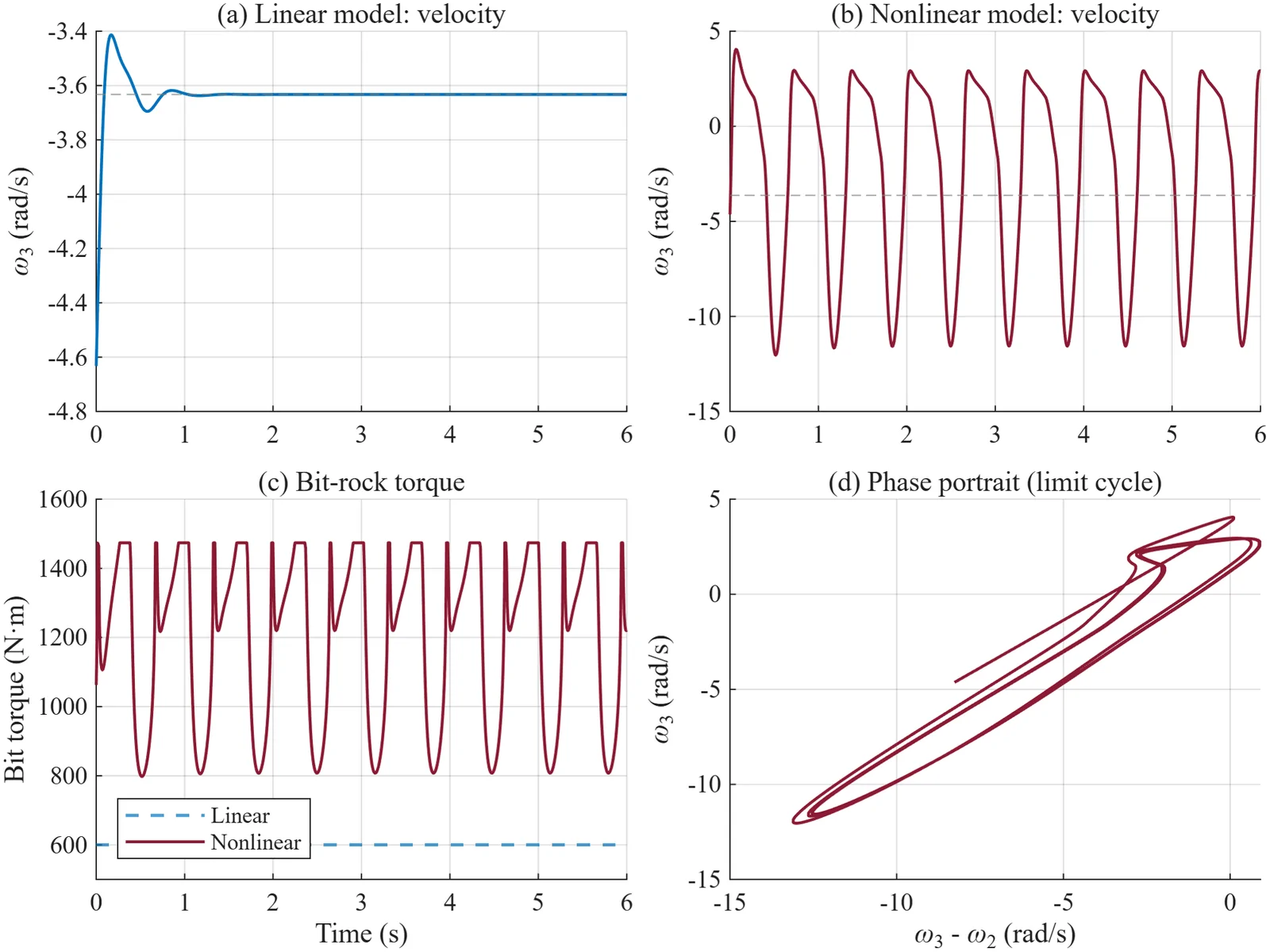
Comparison of stick-slip vibration between linear and nonlinear bit-rock interaction models.

For comparison, the same initial speed disturbance of −1 rad/s is applied to the outer bit, and the results are shown in Fig 8. Under the linear model, the outer-bit speed returns to the original steady state after a short oscillation; under the nonlinear model, the outer-bit speed converges to a new steady state of about 0.80 rad/s after damped oscillation, the bit-rock torque converges to about 2211 N·m, and the phase trajectory converges to a stable equilibrium point without a stick-slip limit cycle. The full-state Jacobian linearization at the equilibrium point of this condition shows that all eigenvalues have negative real parts (the maximum real part is −3.35 1/s), indicating that the system is locally asymptotically stable. The mechanism of the difference between the inner and outer bits lies in: the inner bit is driven in reverse by the lower screw with a transmission ratio η = 2, and the equivalent damping and stiffness converted to the inner-bit side are weakened, so the rate-weakening negative damping is more likely to dominate; while the outer bit is directly coupled with the upper screw motor and the pump-source hydraulic circuit, and can obtain sufficient positive damping. This comparison shows that under the same disturbance and the same friction characteristics, the inner bit is the weak link of stick-slip instability, and the subsequent vibration suppression design and parameter optimization should focus on the inner bit.

**Fig 8:**
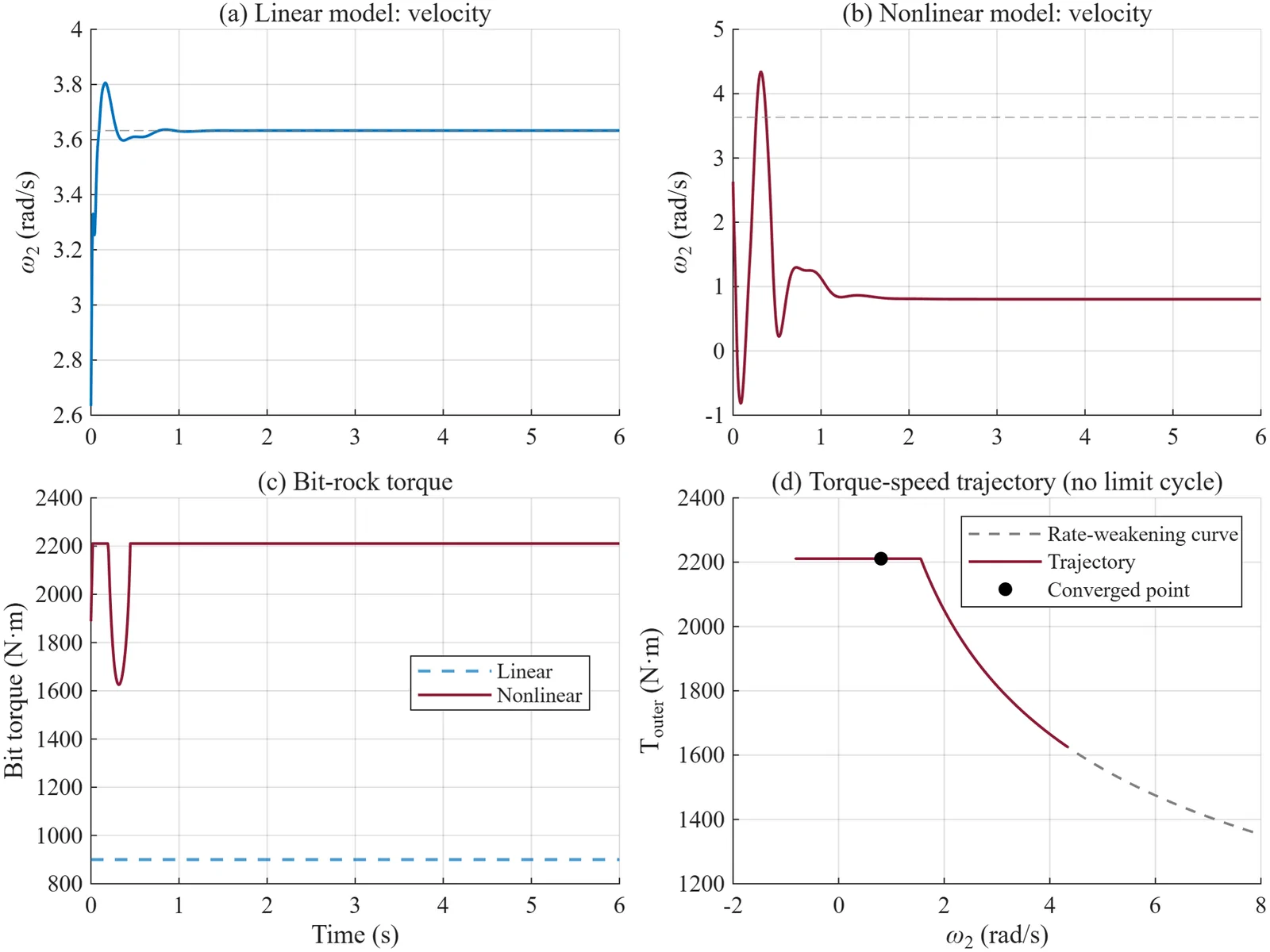
Comparison of stick-slip vibration between linear and nonlinear models for the outer bit.

### 3.4. Vibration suppression effect of the buffer airbag

In deep well drilling, downhole screw drilling tools often withstand severe flow and pressure fluctuations due to sudden load changes. This section deeply discusses the vibration suppression mechanism of the buffer airbag based on the fluid-solid coupling model with airbag.

The buffer airbag is essentially a pneumatic accumulator. When the pressure in the flow path changes suddenly, the inert gas in the airbag changes in volume, absorbs the transient flow, and acts as a “hydraulic low-pass filter.” After introducing the airbag, the fluid network of the system is decoupled into two sections: upstream (pump-upper screw) and downstream (airbag-lower screw), and the order of the state space increases to 9. The comparison of the eigenvalue decomposition results of the improved system with the system without airbag is shown in Table 2.

**Table 2:** Comparison of modal characteristics of the system without and with airbag.

Mode type	Frequency without airbag	Damping ratio without airbag	Frequency with airbag	Damping ratio with airbag	Change of physical meaning
Gas-liquid coupling mode	—	—	about 0.06 Hz	> 1 (overdamped)	New overdamped gas mode (real pole −0.357 1/s, decay time constant about 2.8 s)
Inner-bit-fluid mode	1.76 Hz	0.392	2.13 Hz	0.338	Frequency shifts up, damping basically maintained
Lower-screw fluid-mechanical mode	3.02 Hz	0.350	4.97 Hz	0.566	Hydraulic decoupling, resonance peak greatly weakened
Transmission shaft torsional mode	20.50 Hz	0.291	20.50 Hz	0.291	Basically unaffected by fluid

From the frequency-domain Bode diagram, it can be seen that the airbag reduces the resonance peak amplitude of the inner-bit-fluid coupling near 1.76 Hz by about 3.3 dB (the resonance peak of the lower-screw pressure-drop channel is reduced by about 6.5 dB), effectively isolating the hydraulic impact of the rock-breaking disturbance of the lower bit on the upper screw. The damping ratio of the inner-bit-fluid mode decreases slightly from 0.392 to 0.338, and the damping ratio of the lower-screw fluid-mechanical mode increases from 0.350 to 0.566, enhancing the asymptotic stability of the system.

To quantitatively demonstrate the frequency-domain suppression effect of the airbag, Fig 9 shows the comparison of Bode magnitude characteristics of the inner-bit speed channel and the lower-screw pressure-drop channel under inner-bit load disturbance. It can be seen that with the airbag, the resonance peak of the inner-bit speed channel moves from 1.76 Hz to 2.13 Hz, and its amplitude is reduced by about 3.3 dB; the resonance peak amplitude of the lower-screw pressure-drop channel is reduced by about 6.5 dB, which is consistent with the modal analysis results in Table 2.

**Fig 9:**
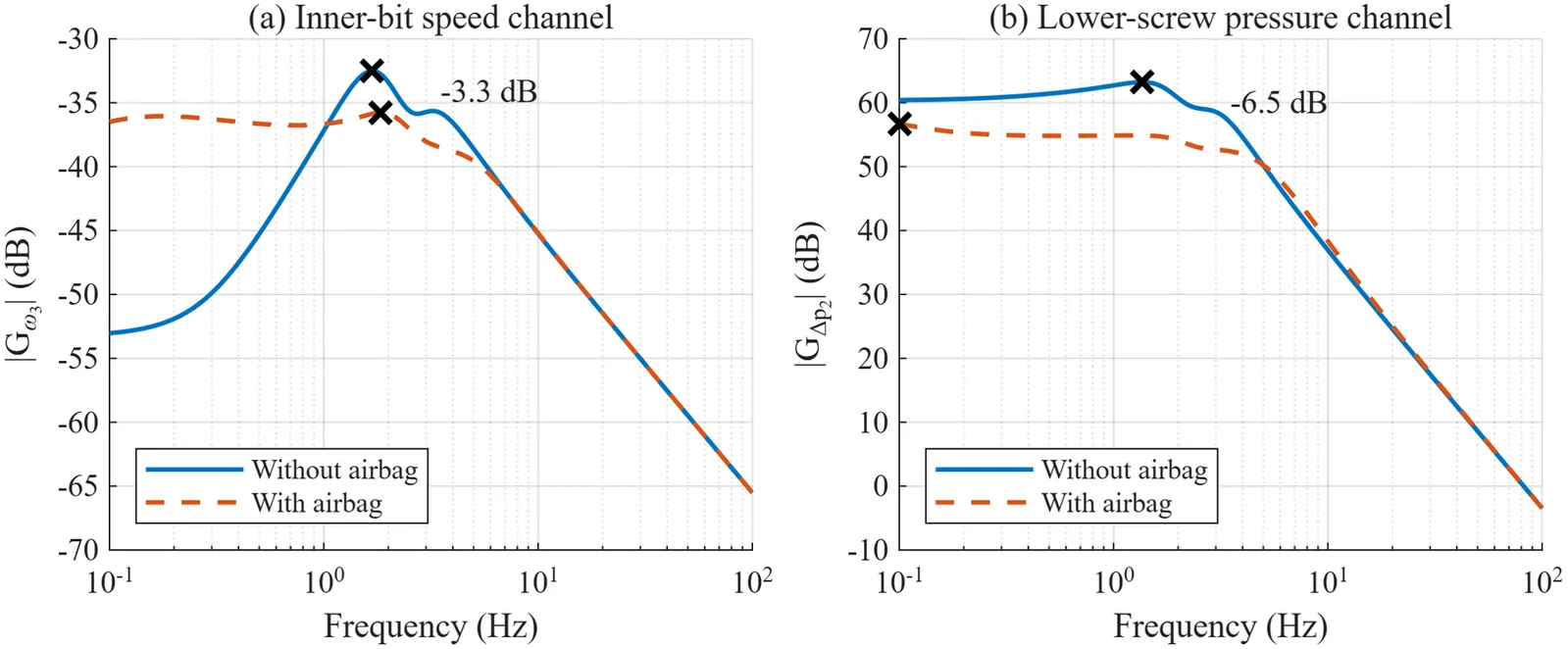
Comparison of frequency-domain Bode magnitude characteristics with vs. without buffer airbag.

(1) Speed overshoot and stability

Under step load, the speed overshoot of the inner bit changes from 8.62% without airbag to 9.73% with airbag, and the influence of the airbag on the step overshoot is limited; its vibration suppression advantages are mainly reflected in impulse load and pressure pulsation conditions, as described below.

(2) Pressure pulsation and stator protection

Under impulse load, the airbag pressure *p*_a_ acts as an intermediate buffer node, so that the Δp_2_ spike is reduced from about 0.228 MPa to about 0.112 MPa, with an attenuation of 51%. The first-peak speed deviation of the inner bit is basically unchanged (2.68 → 3.02 rad/s), but the residual oscillation amplitude after the impact is reduced from 0.548 rad/s to 0.098 rad/s (attenuation of about 82%), and the rising edge of the pressure waveform becomes smooth, effectively protecting the rubber stator of the screw motor.

(3) Nonlinear stick-slip vibration suppression

The nonlinear simulation under the low-speed condition is carried out again. The airbag significantly changes the size of the limit cycle: the half-amplitude of the inner-bit speed oscillation is reduced from 7.247 rad/s to 0.264 rad/s, with an attenuation of 96.4%, and the stick-slip vibration is basically eliminated.

The comparison of the system speed responses with and without the buffer airbag is shown in Fig 10. The comparison of pressure distribution and residual torque with and without the buffer airbag is shown in Fig 11.

**Fig 10:**
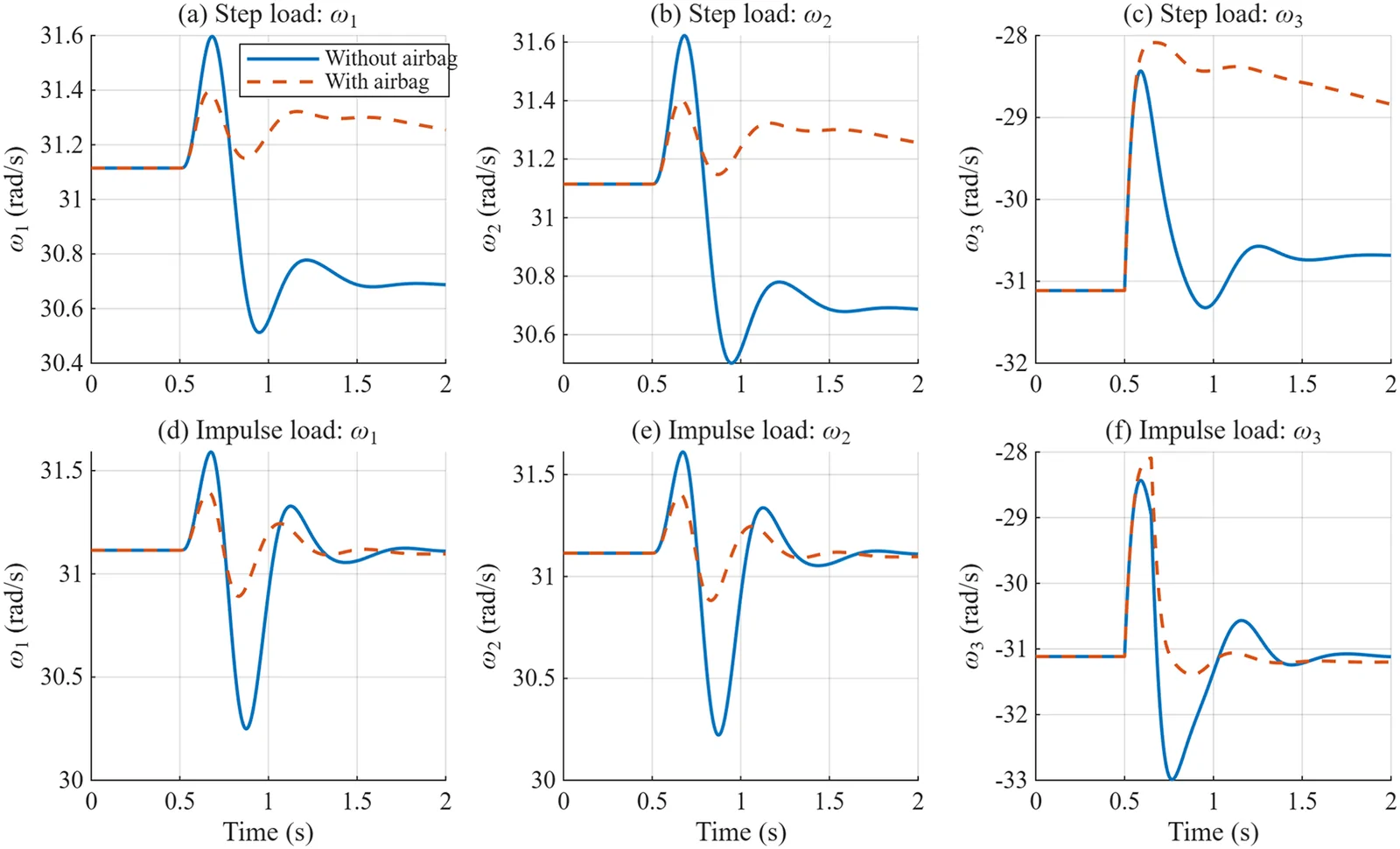
Effect of buffer airbag on angular velocity responses.

**Fig 11:**
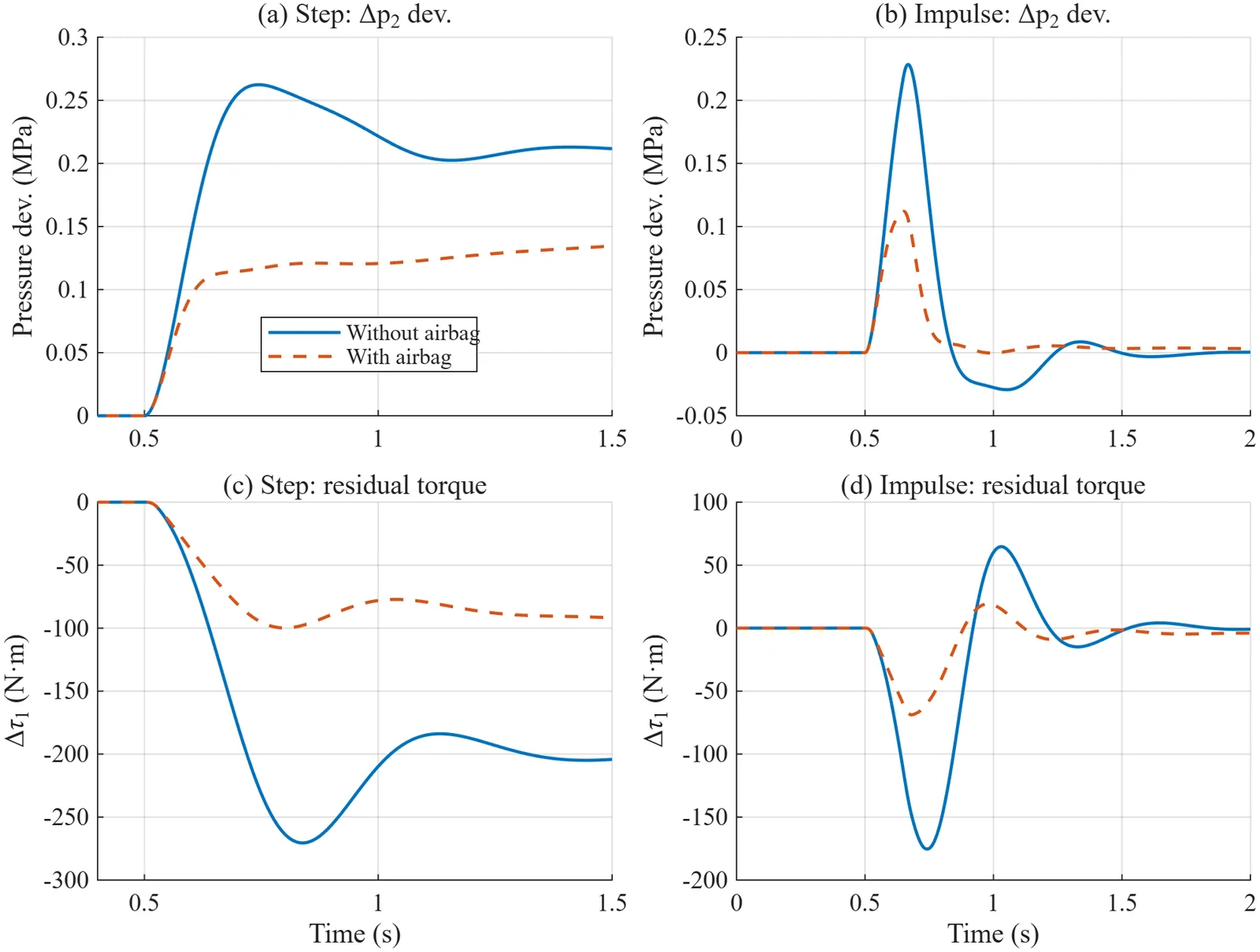
Pressure distribution and residual torque with vs. without buffer airbag.

(4) Evaluation of vibration suppression effect on the outer bit

The same 200 N·m step and impulse loads as in (1) and (2) of this section are applied to the outer bit, and the responses with and without the airbag are compared (Fig 12). The results show that the buffer airbag has almost no effect on the outer-bit side responses: under step load, the maximum speed deviation of the outer bit increases slightly from 0.680 rad/s to 0.689 rad/s, and under impulse load, the upper-screw pressure-drop spike increases slightly from 0.122 MPa to 0.126 MPa. The physical reason is that the airbag is connected to the middle flow path between the upper and lower screw motors, and only absorbs the pressure pulsation of the hydraulic circuit on the inner-bit side (downstream); the outer-bit disturbance is directly constrained by the pump source through the upper screw flow path, and the hydraulic decoupling of the airbag has limited gain for it. This result further shows that the vibration suppression benefit of the airbag is concentrated on the inner-bit side with poorer stability.

**Fig 12:**
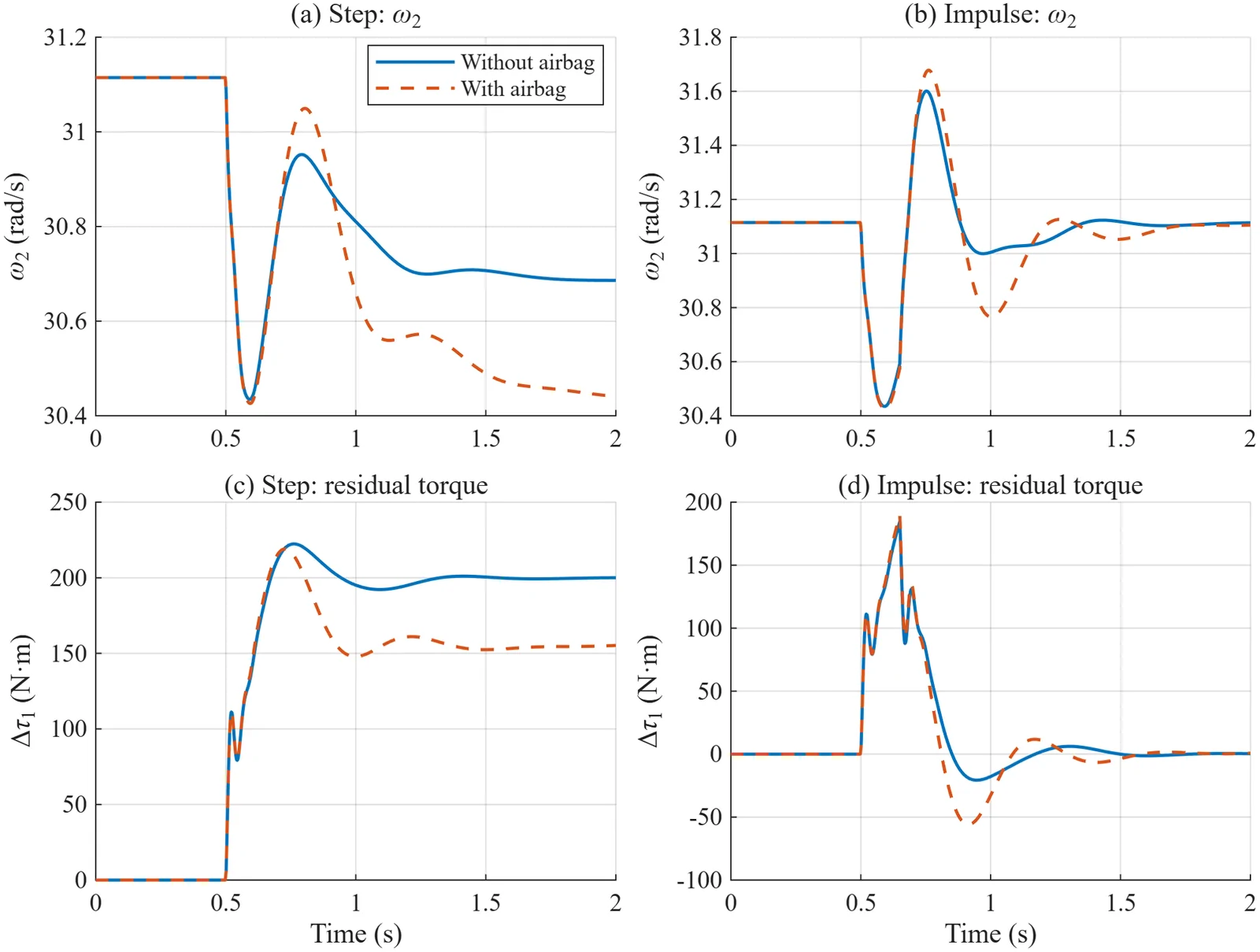
Outer-bit speed and residual torque responses with vs. without buffer airbag.

## 4. Discussion

### 4.1. Optimization of the airbag compliance

In the system with a buffer airbag, in order to further guide the engineering design, this section carries out collaborative optimization and discussion of mechanical-hydraulic parameters based on the control variable method and parameter sensitivity analysis.

The simulation results show that: under equal-amplitude load disturbances, the peak speed deviation of the inner bit (2.68 rad/s) is about four times that of the outer bit (0.680 rad/s), and only the inner bit develops a stick-slip limit cycle under the low-speed condition, indicating that the working stability of the inner bit is significantly poorer than that of the outer bit; the buffer airbag mainly suppresses the pressure pulsation and stick-slip vibration on the inner-bit side (downstream) through hydraulic decoupling, and has limited influence on the outer-bit side responses. Therefore, parameter optimization should aim at improving the working stability of the inner bit.

The airbag compliance *C*_a_ is varied in the range of 10^−9^ to 10^−6^ m³/Pa to evaluate its influence on the overshoot and settling time of the step load response. The results show that: within the whole scanning range, the step overshoot of the inner bit basically remains unchanged at 9.28%−9.84%, which is close to 8.62% without airbag, indicating that the airbag has no direct improvement effect on the step overshoot; the settling time is significantly prolonged with the increase of *C*_a_, from about 2.46 s at 10^−9^ m³/Pa to about 15.8 s at 5 × 10^−8^ m³/Pa, and tends to saturate (about 28–29 s) after the compliance exceeds 10^−7^ m³/Pa. This is because an excessively large airbag compliance introduces a slow gas-liquid coupling mode, which slows down the stabilization process of the system. Therefore, the airbag compliance should not be too large and should take a medium value on the premise of ensuring the pulsation absorption capacity.

The prolonged settling time at large compliance deserves practical attention for field control. The slow convergence is governed by the overdamped gas-liquid coupling mode (real pole −0.357 1/s in Table 2), which primarily affects the slow drift of the mean pressure level, whereas the speed responses relevant to sudden rate-of-penetration shifts or incipient pipe-sticking events are dominated by the much faster 2–5 Hz fluid-mechanical modes and remain well damped. Nevertheless, to bound the transient delay during such events, oversized accumulators (*C*_**a**_ > 10^−7^ m³/Pa, for which the settling time exceeds 25 s) should be avoided; the recommended range 3 × 10^−8^-8 × 10^−8^ m³/Pa keeps the settling time close to that of the baseline design (15.8 s at *C*_**a**_ = 5 × 10^−8^ m³/Pa) while retaining most of the pulsation attenuation. If faster pressure recovery is required during severe sticking events, a smaller airbag combined with active top-drive torque feedback, rather than an oversized passive accumulator, is the preferred engineering solution.

From the perspective of physical implementation, the airbag compliance *C*_a_ = V₀/(mp₀). In the downhole high-pressure environment of 10 MPa, to achieve *C*_a_ = 5 × 10^−8^ m³/Pa, the required initial gas-charging volume V₀ is about 0.7 L. Considering the vibration suppression effect and the limitation of the narrow downhole space on the airbag volume, the recommended design range is 3 × 10^−8^-8 × 10^−8^ m³/Pa, and a design margin of about 30% is reserved for the decrease of effective compliance caused by gas evolution from the drilling fluid.

The sensitivity analysis results of the airbag compliance on the dynamic response of the system are shown in Fig 13.

**Fig 13:**
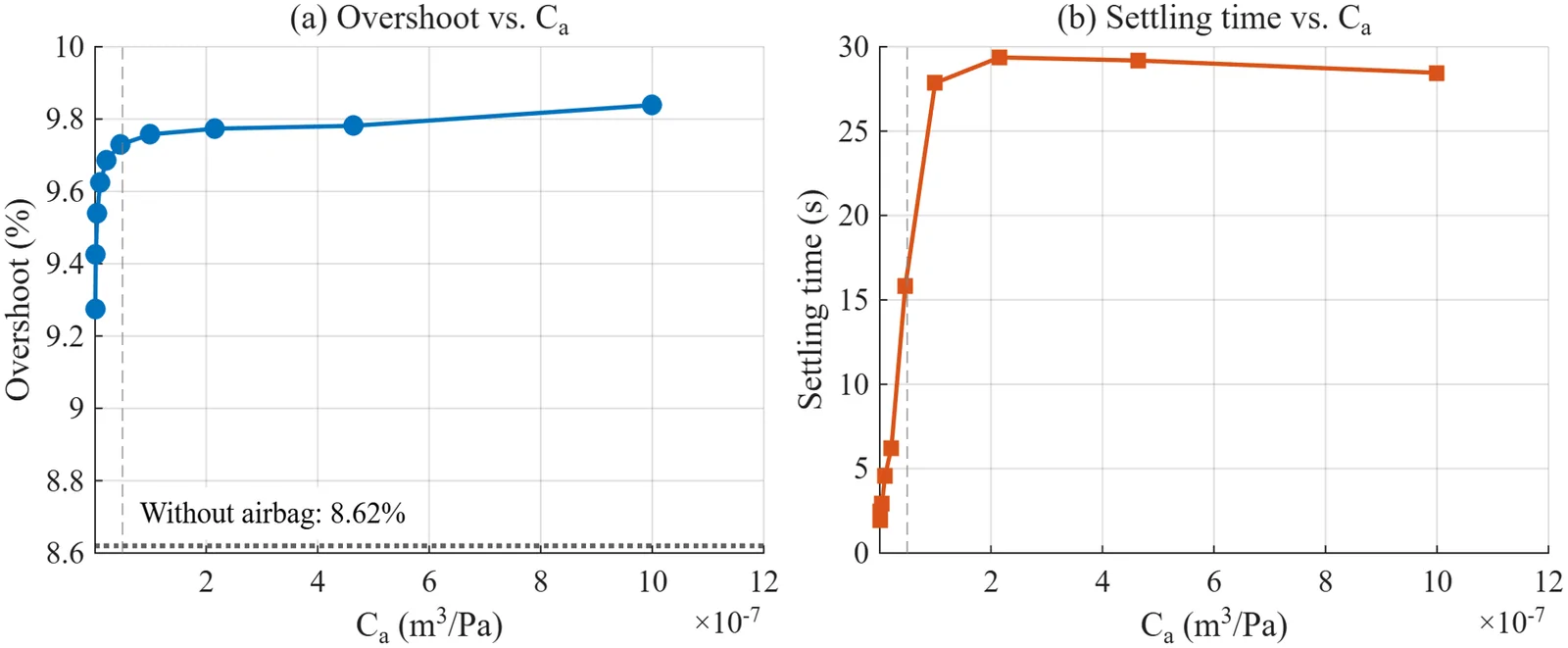
Sensitivity analysis of airbag compliance *C*_*a.*_

### 4.2. Collaborative optimization of mechanical parameters

The influence of mechanical parameters on the overshoot is analyzed by parameter scanning:

(1) Moment of inertia J_3_ (inner-bit inertia) and *c*_d1_ (transmission shaft damping): increasing J_3_ can reduce the step overshoot of the inner bit from about 11.21% (J_3_ = 1 kg·m²) to 9.31% (J_3_ = 6 kg·m²), which is the most effective mechanical parameter means, but it will increase the mass of downhole tools and the difficulty of tripping; increasing *c*_d1_ has a weak effect on the step overshoot dominated by low-frequency fluid (the overshoot basically remains unchanged at 9.73%), and its main function is to enhance the damping of the 20.50 Hz transmission shaft torsional mode, which is still valuable for suppressing torsional fatigue. On the basis of hydraulic decoupling with the airbag, increasing the inner-bit inertia and matching it with a high-damping composite coupling (increasing *c*_d1_) is a direct means to improve the working stability of the inner bit. The free-vibration analysis of rotating components with flexible connections [30] likewise confirms that connection stiffness and contact-state-dependent damping critically govern the modal behaviour of rotating assemblies, supporting the emphasis placed here on matching *c*_**d1**_ and *k*_**1**_.
(2) Transmission shaft stiffness k_1_ and upper screw inertia J_1_: parameter scanning shows that both have weak influence on the step overshoot. When J_1_ increases from 2 kg·m² to 10 kg·m², the overshoot decreases slightly from 9.80% to 9.64%; when k_1_ varies in the range of 2 × 10^4^-1 × 10^5^ N·m/rad, the overshoot basically remains unchanged at 9.72%−9.76%. The selection of k_1_ mainly depends on the separation of modal frequencies: if k_1_ is too low, the torsional modal frequency of the transmission shaft will move down and approach the 2–3 Hz low-frequency fluid-mechanical modes, inducing coupled resonance; it is recommended to take k_1_ = (4–6)×10^4^ N·m/rad to keep the torsional mode in the range of 18–22 Hz, at which the system has the best robustness to the change of *c*_d1_.

### 4.3. Working stability analysis and optimization of the inner bit

Based on the simulation results in Section 3, under equal-amplitude load disturbances, the peak speed deviation of the inner bit (2.68 rad/s) is about four times that of the outer bit (0.680 rad/s), and only the inner bit develops a stick-slip limit cycle under the low-speed condition, indicating that the working stability of the inner bit is significantly poorer than that of the outer bit. The internal reasons can be attributed to the following three points:

(1) Kinematic amplification and inertia mismatch: the inner bit is driven in reverse by the lower screw with a displacement ratio η = 2, the driving torque and speed response converted to the inner-bit side under the same pressure-drop disturbance are amplified, and the inner-bit inertia J_3_ is the smallest, so the anti-disturbance ability is weak;
(2) Weakening of equivalent damping: the viscous damping c_3_ of the inner-bit-lower-screw-rotor circuit is relatively low relative to its inertia and converted stiffness, and the rate-weakening negative damping is more likely to dominate under the low-speed condition, forming a stick-slip limit cycle;
(3) Hydraulic boundary constraint: the outer-bit disturbance can be directly transmitted to the pump source through the upper screw flow path and be constrained, while the inner bit is at the end of the series circuit, and the pressure pulsation is reflected and superimposed at the end, further aggravating its oscillation.

Aiming at the above reasons, the following optimization suggestions focusing on the inner bit are proposed: (1) appropriately increase the inner-bit inertia J_3_ and match it with a high-damping composite coupling (increasing *c*_d1_), which can reduce the step overshoot of the inner bit from about 11.21% to 9.31%; (2) increase the bearing damping c_3_ on the inner-bit side to improve the equivalent damping ratio of the inner-bit circuit and suppress the low-speed stick-slip oscillation; (3) install a buffer airbag in the middle of the series flow path (recommended compliance *C*_a_ = 5 × 10^−8^ m³/Pa, corresponding to an initial gas-charging volume V₀ ≈ 0.7 L required at a downhole pressure of 10 MPa), and use hydraulic decoupling to absorb the pressure pulsation on the inner-bit side, which can attenuate the impulse pressure-drop spike by 51%, the residual oscillation after impact by about 82%, and the stick-slip limit-cycle amplitude by more than 96%.

### 4.4. Field application considerations

The theoretical analysis of this study shows that the series architecture of positive- and reverse-hand screw motors combined with the middle airbag can significantly improve the downhole dynamic characteristics. However, the following issues still need to be paid attention to in practical engineering applications:

(1) Sealing and strength reliability: the adapter assembly needs to withstand high-pressure fluid and alternating torsional stress at the same time, and the sealing design of the airbag container is the key. For the fatigue evaluation of such alternating-load downhole structures, the decoupling-based fatigue life assessment methodology developed for subsea wellhead systems [31] provides a transferable framework, in which global dynamic loads are mapped to local hot-spot stresses for S-N-based life prediction.
(2) Gas dissolution problem: under high pressure, nitrogen may gradually dissolve into the drilling fluid, resulting in a decrease in airbag volume (compliance reduction). The feasibility of using a piston accumulator isolated by hydraulic oil needs to be studied in the future. The consequence of this dissolution can be quantified from the sensitivity analysis in Fig 13: a gradual loss of gas volume shifts the effective compliance *C*_**a**_ downward from its design value, which progressively weakens the pressure-pulsation attenuation—the dominant benefit of the airbag—while leaving the step overshoot essentially unchanged (9.28%−9.84% over the full scan). A design margin of about 30% on the initial gas charge is therefore reserved (Section 4.1), and the nitrogen charge pressure should be inspected and restored at each scheduled overhaul of the screw motor (a PDM motor typically operates for about 200 h under high-torque conditions between overhauls). For long-duration operations, a piston-type or bladder-type accumulator, in which the gas is physically isolated from the drilling fluid, eliminates the dissolution path entirely, at the cost of a more complex downhole structure.
(3) Temperature influence: the high temperature at the bottom hole will lead to the change of the gas polytropic index, and *C*_a_ needs to be corrected according to the temperature profile in actual design. Recent work on active wellbore cooling with microencapsulated phase-change materials [32] shows that the bottomhole circulating temperature can be actively reduced (by up to about 21 °C under their baseline conditions), offering a complementary means to stabilize the gas polytropic behaviour of the airbag in deep, hot wells.
(4) Durability of the high-damping composite coupling: the composite coupling recommended in Section 4.2 for suppressing the 20.50 Hz torsional mode must survive the downhole thermal and torsional environment over extended drilling hours. Elastomer-based damping elements (NBR/HNBR compounds) provide the highest specific damping but undergo thermal aging above roughly 120–150 °C, which hardens the material, degrades damping and eventually causes cracking; FKM/FFKM grades extend the service temperature at higher cost. Metal-based alternatives (disc-spring or wire-rope damping elements with friction interfaces) retain their properties beyond 200 °C and tolerate higher transmitted torque, at the expense of lower inherent damping. In field practice, the coupling should be derated so that the transmitted torque stays within 60–70% of its rated capacity, and its damping elements should be inspected or replaced together with the screw-motor elastomer stator at each overhaul (about 200 h of high-torque operation). A hybrid scheme—a metal coupling body with replaceable high-temperature elastomer inserts—is therefore recommended, and its thermal-fatigue qualification testing is planned as future work.
(5) Dynamic weight on bit and axial-torsional coupling: the present model treats WOB as quasi-static and omits bit rebound. In hard formations, axial bit bounce modulates the instantaneous WOB and hence the reference rock-breaking torque *T*_**ref**_ in Equation (20), which can strengthen or weaken the effective rate-weakening negative damping. This simplification is acceptable for the present scope because the WOB regulation bandwidth at the rig (typically below 0.1 Hz) is well separated from the dominant torsional-hydraulic modes (1.76–20.50 Hz), and the airbag hydraulically decouples the path that would otherwise transmit pump-pressure fluctuations to the bits. A fully axial-torsional coupled extension—in which *T*_**ref**_ becomes a function of the instantaneous WOB and the bit axial dynamics are added as extra states—is a priority direction for future work.
(6) High-frequency pressure waves and localized nozzle losses: Equations (11)-(13) lump the fluid inertance and resistance into constant parameters. This is valid while the pressure-wave transit time along the tool flow path is much shorter than the period of the fastest dynamic mode: for a tool-length flow path of about 10 m and a sound speed of 1000–1200 m/s in water-based drilling fluid, the transit time is about 10 ms (characteristic frequency near 100 Hz), well above the fastest torsional mode (20.50 Hz), so wave-propagation effects do not alter the reported dynamic states. The localized quadratic losses of the bit nozzles are quasi-steady on the millisecond scale: they shift the steady operating point (absorbed into the calibration of *R*_**f**_ at the rated flow) and their linearized slope adds positive damping, which is slightly conservative for stability predictions. A distributed-parameter hydraulic model with explicit nozzle orifices will be incorporated in future high-fidelity extensions.

## 5. Conclusions

In this paper, a systematic study on coupled dynamics modeling and vibration suppression of a torque-balanced drilling tool driven by positive- and reverse-hand screw motors in series is carried out. The main conclusions are as follows:

(1) Verification of torque balancing mechanism: through the displacement matching of the twin screw motors in series (η = 2) and the mechanical connection of the transmission shaft, the reverse rotation of the outer bit and the inner bit with equal speed and opposite direction is realized. The residual drill-string torque in steady state is only 300.0 N·m, which is 66.7% lower than that of the traditional single-bit drilling tool (900 N·m).
(2) Revelation of the dynamic characteristics of the system without airbag: modal analysis identifies three dominant oscillation modes (1.76 Hz, 3.02 Hz, 20.50 Hz), with damping ratios between 0.291 and 0.392, belonging to a weakly damped system. The inner bit is the most sensitive to disturbances due to its small inertia and large transmission ratio, with a speed overshoot of 8.62% under step load; under equal-amplitude disturbances, the peak speed deviation of the inner bit is about four times that of the outer bit; under the low-speed condition, only the inner bit develops a stick-slip limit cycle (peak-to-peak value 14.5 rad/s), while the outer bit converges to a new steady state after disturbance, indicating that the working stability of the inner bit is significantly poorer than that of the outer bit.
(3) Nonlinear effects: the bit-rock interaction considering rate-weakening introduces negative damping (*c*_eq_ < 0), which makes the system prone to stick-slip self-excited vibration; the Jacobian at the nonlinear equilibrium point of the inner bit has eigenvalues with positive real parts (2.25 1/s and 53.18 1/s), while all eigenvalues of the outer-bit nonlinear equilibrium point have negative real parts (maximum real part −3.35 1/s), indicating that the inner bit is the weak link of stick-slip instability.
(4) Vibration suppression effect of the buffer airbag: after installing a buffer airbag with a compliance *C*_a_ = 5 × 10^−8^ m³/Pa, the system achieves hydraulic decoupling. The pressure-drop spike under impulse condition is attenuated by 51% (0.228 MPa → 0.112 MPa), the residual oscillation amplitude after impact is attenuated by about 82% (0.548 rad/s → 0.098 rad/s), and the half-amplitude of the stick-slip limit cycle under the low-speed condition is reduced by 96.4% (7.247 rad/s → 0.264 rad/s).
(5) Parameter optimization guidance: when the airbag compliance exceeds 10^−7^ m³/Pa, the settling time is significantly prolonged and tends to saturate; increasing the inner-bit inertia (overshoot reduced from about 11.21% to 9.31%) is the most effective mechanical means to suppress the overshoot; the transmission shaft stiffness is recommended to be (4–6)×10^4^ N·m/rad.

This study provides a theoretical basis for the structural optimization and field application of torque-balanced drilling tools. Future work will consider the distributed-parameter model of the drill string, axial-torsional coupled vibration, and the change of fluid physical properties under high-temperature and high-pressure environments.

## Supporting information

S1 File Python simulation code. Author-generated Python script implementing the coupled torsional-hydraulic model, the steady-state operating-point solution, the modal and frequency-domain analyses, and all time-domain transient simulations reported in this study (Figs 3–13 and Tables 1–2).
(PY)

S2 File Data (simulation results). Author-generated numerical dataset containing the simulation results underlying Figs 3–13 and Tables 1–2, including steady-state operating points, modal frequencies and damping ratios, frequency-response data, time-domain responses under step and impulse loads, nonlinear stick-slip results, and parameter-sensitivity analysis data.
(XLSX)

## Abbreviations

PDM: Positive displacement motor
WOB: Weight on bit
BHA: Bottomhole Assembly
ROP: Rate of penetration
HFTO: High-frequency torsional oscillation
